# The hologenome concept of evolution after 10 years

**DOI:** 10.1186/s40168-018-0457-9

**Published:** 2018-04-25

**Authors:** Eugene Rosenberg, Ilana Zilber-Rosenberg

**Affiliations:** Department of Molecular Microbiology and Biotechnology, Tel Aviv University, Ramat Aviv, Israel

## Abstract

The holobiont (host with its endocellular and extracellular microbiome) can function as a distinct biological entity, an additional organismal level to the ones previously considered, on which natural selection operates. The holobiont can function as a whole: anatomically, metabolically, immunologically, developmentally, and during evolution. Consideration of the holobiont with its hologenome as an independent level of selection in evolution has led to a better understanding of underappreciated modes of genetic variation and evolution. The hologenome is comprised of two complimentary parts: host and microbiome genomes. Changes in either genome can result in variations that can be selected for or against. The host genome is highly conserved, and genetic changes within it occur slowly, whereas the microbiome genome is dynamic and can change rapidly in response to the environment by increasing or reducing particular microbes, by acquisition of novel microbes, by horizontal gene transfer, and by mutation. Recent experiments showing that microbiota can play an initial role in speciation have been suggested as an additional mode of enhancing evolution. Some of the genetic variations can be transferred to offspring by a variety of mechanisms. Strain-specific DNA analysis has shown that at least some of the microbiota can be maintained across hundreds of thousands of host generations, implying the existence of a microbial core. We argue that rapid changes in the microbiome genome could allow holobionts to adapt and survive under changing environmental conditions thus providing the time necessary for the host genome to adapt and evolve. As Darwin wrote, “It is not the strongest of the species that survives but the most adaptable”.

## Background

Ten years ago, we introduced the hologenome concept, which considers the holobiont with its hologenome as an independent level of selection in evolution [1, 2]. During the last few years, the hologenome concept of evolution has received considerable support and legitimate criticism, both of which have enriched the concept and led to a better understanding of what constitutes an organism, and how it evolves.

Regarding definitions, the term holobiont, first introduced by Margulis in 1991 [3], now refers to an animal or plant host together with all associated microorganisms living on or in it, exosymbionts and endosymbionts, respectively. The microbiome refers to all of the microbes associated with an animal or a plant [4]. The term microbiota preceded the term microbiome and by some is considered synonymous to it. In this paper, we refer to microbiota as being the microbes associated with an animal or plant, but not necessarily the entire community of microbes. The union of all the genes in the holobiont, i.e., all the genes in the microbiome plus the genes of the host, constitutes the hologenome [1, 2]. The definition of a species for bacteria is controversial [5]. The most widely used bacterial species definition is a group of strains showing over 97% of 16S rDNA gene-sequence identity [6].

The hologenome concept of evolution was, and still is, based on accumulated findings, from which we have extracted four basic principles:

1. All animals and plants harbor abundant and diverse microbiota and are thus considered holobionts.
2. The host with its microbiome, the holobiont, functions generally as a distinct biological entity anatomically, metabolically, immunologically, during development and in evolution. (An entity is defined as “an independent thing; that which contains in itself the conditions essential to individuality; that which forms of itself a complete whole.”)
3. A significant fraction of the microbiome genome together with the host genome is transmitted from one generation to the next and thus can propagate unique properties of the holobiont.
4. Genetic variation in the hologenome can be brought about by changes in the host genome as well as by changes in the microbiome genome. Since the microbiome genome can adjust to environmental dynamics more rapidly and by more processes than the host genome, it can play a fundamental role in the adaptation and evolution of the holobiont.

The hologenome concept considers all holobionts to exist on a spectrum from extreme symbiosis (obligatory) to a looser state of symbiosis. The endosymbionts with their host are usually an example of an extreme case of mutual metabolic and genetic adaptation with clear vertical transmission. Humans with their exosymbionts, however, are an example of a much more complex and seemingly looser symbiosis and mode of transmission; however, in both cases, the fitness (and in many cases survival) of most of the holobionts tested to date depends on the mutual interactions between all of its participants and on reasonably accurate transmission of the microbiota.

In this review, based on recent experimental and theoretical research, we discuss the current status of each of the four principles of the hologenome concept, their pros and cons, and thereby derive a fuller picture of the evolution of holobionts with their hologenomes.

## All animals and plants are holobionts

The initial generality that all natural animals and plants contain abundant and diverse microbiotas has now been substantiated by analyses of numerous organisms (Table 1). However, as will be discussed below, the complexity and dynamics of microbiomes are only beginning to be appreciated.

**Table 1:** Numbers of bacterial species associated with animals and plants: examples

Host	Number of bacterial species	Reference
Invertebrates		
*Drosophila*	209	Wong et al. [183]
Marine sponge	2996	Schmitt et al. [184]
Coral	2050	Ainsworth et al. [185]
Honey bee	336	Moran et al. [186]
Termite gut	800	He et al. [187]
Nemotode *C. elegans*	87	Dirksen et al. [188]
Butterfly. *H. erato*	45	Hammer et al. [189]
Vertebrates		
Human gut	5700	Nam et al. [190]
Human skin	1000	Ying et al. [191]
Bovine rumen	5271	Jami and Mizrahi [192]
Great ape gut	8914	Ochman et al. [124]
Cottonmouth snake	503	Colston et al. [193]
Marine iguana	896	Hong et al. [194]
Folivorous flying bird	580	Godoy-Vitorino et al. [195]
Panda gut	781	Xue et al. [196]
Plants		
Rice		Edwards et al. [197]
Alga *Ulva australis*	642	Burke et al. [198]
Carnivorous plant	1000	Koopman et al. [199]
Arabidopsis	8000	Bai et al. [200]
Phyllosphere	87	Bulgarelli et al. [78]
Oak rhizosphere	5619	Mendes et al. [201]

The last few years have witnessed calls for major national and international efforts to characterize holobiont microbiomes [7, 8]. Knowledge about microbiomes has come primarily from studies on bacteria of the human and ruminant gut, but many more hundreds have been examined to date. From the published information, several generalizations have emerged that enable better understanding of what determines abundance and diversity and, as a result, what determines the relationship between the microbiome and its host.

### Quantitative considerations

The human gut contains approximately 4 × 10^13^ bacteria, similar to the total number of human cells in the body [9]. Because of the large diversity of bacterial species, the gut microbiome contains ca. 9 million unique protein-coding genes or 400 times more bacterial genes than human genes [10]. It is important to realize that the bacterial count of the different bacterial species can differ by several orders of magnitude. In some cases, as few as 40 abundant bacterial species accounts for ca. 75% of the human gut microbiome [11]. However, bacterial species that are present at less than 4 × 10^7^ copies (0.00001% of the total) cannot be detected by current methods. Such relatively rare bacterial species should not be ignored since they have the potential to amplify under different conditions and play a role in adaptation and evolution of the holobiont. Clearly, analyses of the taxonomic diversity associated with the human microbiome will continue to be an area of great importance.

The gut microbiome also contains abundant and diverse viruses [12] and fungi [13], but they have not been studied extensively.

### Individual variation: core and conserved function

High-throughput sequencing has demonstrated that although the bacterial species composition within the human gut is unique to each person, microbiomes of different individuals are closer to each other than to microbiomes of other primates [14]. These data suggest that there is something common (a core) to the human microbiome. Part of the Human Microbiome Project (HMP) has provided an opportunity to examine and better define what constitutes the taxonomic core within and across body habitats and individuals [15]. Shapira [16] has emphasized the differences between conserved core microbiota and flexible, environmentally driven microbiota with regard to their maintenance and contributions to host adaptation. However, attempts to identify “core” bacterial species in the gut microbiome have yielded only a few common species. Nonetheless, as discussed above, the presence or absence of a bacterial species depends on technical limits of detection. Methods developed to detect rare species may reveal that there are many more common species than currently considered and that individual variation may be the result of quantitative rather than qualitative differences that are caused by a different diet or some other environmental factor.

An alternative explanation is possible. Although human gut microbiomes vary between individuals in taxonomic composition, the biological functions they perform are surprisingly invariable between different people. Humans harbor phylogenetically distinct gut communities that can carry out the same functions [17, 18]. This means that there is considerable metabolic redundancy, genes, or isogenes for the same function being distributed across many species, so that a healthy gut microbiome can be assembled in many ways [19]. This idea was expressed in 2009 by Turnbaugh et al. [20] and lately in an article by Doolittle and Booth [21], titled: “It’s the song, not the singer….”. This is consistent with the hologenome concept, which considers all of the genes of a holobiont, not necessarily specific species.

### Variation with time and environmental conditions

The composition of human gut microbiomes and their corresponding hologenomes change with age, diet, medication, and many other factors. Gut microbiomes of newborns are dominated by facultative anaerobes such as the Proteobacteria, after which the diversity of strict anaerobes within the Firmicutes and Bacteroidetes phyla increases towards an adult-like profile by approximately 1 year of age [22]. Throughout this early developmental stage, microbial composition is shaped by mode of delivery [23], infant diet [24], antibiotic treatment [25], and exposure to environmental factors, such as furry pets [26]. During most of adult years, the microbiome seems to be more or less stable [27]. The gut microbiome in older people (> 65 years), however, is extremely variable between individuals and differs from the microbiome of younger adults [28]. Microbiomes of people in long-stay care centers are less diverse than that of community dwellers and are correlated with low fiber diets and increased frailty [29].

Food, food additives, and essentially any material that is put in the mouth affects the gut microbiota at all ages. Both long-term [30] and short-term [31] diet influences the human gut microbiota. An “animal-based diet,” rich in meats, eggs, and cheeses increases the abundance of bile-tolerant microorganisms (Alistipes, Bilophila, and Bacteroides), whereas a “plant-based diet,” composed of grains, legumes, fruits, and vegetables, increases the levels of Firmicutes that metabolize dietary plant polysaccharides. Not only macronutrients, but also other components that are consumed affect the microbiome, such as red wine [32], tea and coffee [33], chocolate [34], food emulsifiers [35], artificial sweeteners [36], and, of course, antibiotics [37]. Also, the microbiome is affected by other factors such as, physical activity [38] and illnesses, e.g., cancer [39] and diabetes [40].

### Host genetics affects the microbiome

In addition to environmental factors, host genetics plays a role in the acquisition, maintenance and stability of gut microbiota [41–43]. The three components, environment, host genetics, and microbiome, interact to maintain homeostasis in the gut. The disruption of this stability by modifying one or more of these three interacting components may trigger the development of diseases. It has been shown that a single host gene can have a large effect on the diversity and population structure of the gut microbiota. Most of the genes shown to have an impact on the composition of the gut microbiome are components of the immune system.

### Endosymbionts and exosymbionts

Although exosymbionts are present in all animals and plants, it is well documented that non-pathogenic endosymbiosis is common only among plants and invertebrates (e.g., insects and corals). To the best of our knowledge, endosymbionts are not present in vertebrates. Invertebrates and plants have developed homeostatic interactions with the endosymbionts to benefit both. Moreover, they have developed specific immunological systems that participate in maintaining and controlling this important relationship [44, 45]. The close proximity of endosymbionts to host nuclei may enhance exchange of genetic material, as discussed in the section on “Genetic variation and evolution of holobionts.” In vertebrates, the more complex immune system appears to limit penetration of microorganisms into organs and cells [46, 47], while enabling a homeostatic and often beneficial relationship to develop with the exosymbionts.

## Interactive fitness in holobionts

Since the original presentation of the hologenome concept of evolution [2], a large number of studies have demonstrated the beneficial interactions between microbiomes and their hosts, leading to a better-adapted holobiont. In obligatory symbioses, the interdependence between host and microbiome is absolute. In many other holobionts, the measure of interdependence of the participants differs. Though it is not always easy to demonstrate the effect of the microbiome on survival and reproduction with facultative symbionts, it has been demonstrated in a number of systems. Stunted growth, shortened lifespan, and deteriorating reproduction were demonstrated in water fleas [48], termites [49], and firebugs and cotton strainers [50]. However, a large volume of data has demonstrated that microbiomes participate in many functions within the holobiont, as will be described immediately, though the extent of their requirement is not always clear.

### Protection against pathogens

In general, germfree (GF) animals are more sensitive to infection by pathogens than conventional (CV) animals [51]. Following oral infection, the numbers of *Listeria monocytogenes*, a pathogenic bacteria, were 10,000-fold higher in the small intestine of GF mice compared to CV mice [52]. *Staphylococcus aureus* infection is prevented by resident *Corynebacterium* species [53]. Recently acquired symbiotic bacteria protect corals against the bleaching pathogen *Vibrio shiloi* [54]. Production of antibiotics is a common mechanism by which resident bacteria protect the holobiont against pathogens [55]. One of the strongest arguments for the role of bacteria in combatting infectious disease is the successful treatment of patients, suffering from severe diarrhea caused by *Clostridium difficile* infection, with fecal transplants from healthy donors [56]. Bacteria have also been shown to protect plants against infectious diseases by inhibiting the phytopathogen and by inducing systemic resistance [57, 58].

### Provision of nutrients

An important general fitness contribution of microbiomes to their hosts is carrying out metabolic processes that the animal or plant cannot carry out by themselves [59]. There are many examples: nitrogen fixation in legumes [60], cellulose degradation in ruminants [61], termites [62] and cockroaches [63], essential amino acid synthesis in insects [64], photosynthesis by microalgae in corals, mollusks and sponges [65], and oxidation of inorganic compounds [66] and hydrocarbons in deep-sea invertebrates [67].

In humans, gut bacteria have been shown to perform several beneficial biochemical reactions that cannot be carried out by the host. For example: (i) production of metabolites from dietary components, such as the conversion of dietary fiber to the short-chain fatty acids, acetate, propionate. and butyrate [68]; (ii) modification of metabolites that are produced by the host, such as primary bile acids that are converted to secondary bile acids, thus assisting in bile acid recycling [69]; (iii) de novo synthesis of compounds, such as the important microbial immune modulator polysaccharide A, produced by the common gut bacterium *Bacteroides fragilis* [70]; (iv) synthesis of vitamins. Certain gut bacteria can produce vitamin K as well as most of the water-soluble B vitamins [71].

### Fat storage and obesity

Not only is the composition of the gut microbiome of obese and lean individuals different [72], but more significantly, fecal bacteria transferred from obese humans to germ-free mice caused a greater increase in body weight than transplants from lean humans [73], suggesting that the microbiome in combination with diet and genetic factors causes obesity. Although bacteria that contribute to obesity could be considered harmful, under certain conditions they are beneficial. During the third trimester of pregnancy, these so-called “obese bacteria” become abundant and induce metabolic changes that promote energy storage in fat tissue that in turn encourages growth of the fetus [74] and milk production in the mother. Also, during our evolution, food insecurity was a frequent occurrence, and the ability to store energy in the form of fat was probably advantageous for survival.

### Development and behavior

It is has been known for many years that certain microbial symbionts (once termed primary symbionts) interact with their hosts to benefit the holobionts: Rhizobia strains cooperate with legume plants to produce root nodules that perform nitrogen fixation [75]. *Vibrio fisheri* triggers the formation of the light organ in squid, where luminescence occurs to help the squid avoid predation [76]. Intracellular algae of the genus *Symbiodinium* carry out photosynthesis that provides nutrients to their host coral [77]. In recent years, it has been shown that co-development of the microbiome with animals and plants is not limited to primary symbionts. In plants, microbes associated with root tips acquire nutrients from plant secretions and in turn produce indole acetic acid that stimulates root elongation and lateral root formation [78]. In vertebrates, the gut microbiome promotes the development of the immune system and body organs [79]. Exposure to microorganisms educates the immune system, induces innate and adaptive immunity [80, 81], and initiates memory B and T cells that are essential to combat various pathogens. In addition, the gut microbiome encourages also the development of bone mass [82] and blood vessels in the intestinal wall [83].

*Hydra* is an evolutionarily ancient multicellular organism which has been used as a model system in developmental biology. Recently, it has been shown that *Hydra* microbiota plays an essential function in reproduction [84] and influences the spontaneous contractions, likely by modulating the pacemaker activity movement [85].

Bacteria in the mammalian gut also modulate brain development and behavior, including anxiety and mood disorders [86]. Data from experiments performed in rodents with altered intestinal microbiota, whether germ-free mice, or conventionally raised animals treated with probiotics and/or antibiotics, all indicate that rodent behavioral responses are impacted when the bacterial status of the gut is manipulated [87]. Microbial gut–brain signaling is bidirectional. The circuitry of neurons, hormones, and chemical neurotransmitters allows messages to be transmitted between the brain and the gut. For example, the rate at which food is being moved and how much mucus is lining the gut—both of which can be controlled by the brain—have a direct impact on the environmental conditions the microbiota experiences. On the other hand, the gut microbiota influences the body’s level of the potent neurotransmitter serotonin, which promotes in addition to gut functions also feelings of happiness and peacefulness [88].

### Microbiomes warm their hosts

Recently, we suggested that provision of heat is an underappreciated general contribution of microbiomes to holobionts [89]. Microbiomes produce heat as a by-product of the enzymatic catabolism of substrates and synthesis of cell material. It was reported that bacteria have specific rates of heat production of ca. One hundred sixty-eight milliwatts per gram [90, 91]. Based on these findings, it can be calculated [92] that about 70% of human body heat production at rest is the result of bacterial metabolism in the gut.

Consistent with the concept that microbes warm their hosts are reports that treatment of rabbits [93] and rodents [94] with antibiotics lowered their body temperature. Heat output by gut microbiota may also help explain the observation that germ-free mice had 40% less total body fat than conventionally raised mice, even if their caloric intake was 29% higher [95]. The warming effect of microbiomes has also been reported in plants [96]. Heat produced by the sugar catabolism of yeast populations inhabiting floral nectar increased the temperature of the nectar and modified the within-flower thermal microenvironment.

Though the significance of heat production by microbiomes has scarcely been studied, its contribution may have far-reaching implications. It may help warm-blooded animals avoid hypothermia, and in cold-blooded animals, it can raise their body temperature.

## Transmission

For holobionts to be considered units of selection in evolution, both the host and microbiome genomes, i.e., the hologenome, are expected to be transferred between generations. The conservative mechanism for transmission of host DNA is well understood and need not be discussed here. Transmission of the microbiome also occurs, but with a variety of mechanisms and with less precision than the host genome. This section will describe the different modes of microbiome transmission and the evidence that it is transferred for many generations. Table 2 summarizes examples of the main modes of microbiome transmission.

**Table 2:** Examples of modes of symbiont transmission

Mode of transmission	Examples
Vegetative reproduction (vertical)	Plants, worms [99], corals [202], echinoderms [203]
Via oocytes (vertical)	*Drosophila*/*Wolbachia* [98], aphid/*Buchnera* [97], sponge [204], herbs/fungi [205]
Coprophagy (vertical and horizontal)	Many animals: termites [101], rabbits [206], koala [100], insects [102]
Mother’s milk (vertical)	Mammals [108–110]
Physical contact starting at birth (Vertical and horizontal)	Most animals: fish [207], amphibians [208], mammals [209]
Horizontal	Grasses/endophytes [120], squid/*Vibrio fischeri* [119]

Adapted from Roughgarden et al. [182]. Vertical transmission is defined as the movement of microbiota from parent to offspring without mixing with microbes in the environment

Vegetative reproduction occurs in many plants and some animals. In plants, this type of asexual reproduction can involve adventitious roots, corms, tubers, bulbs, and leaf plantlets. Vegetative reproduction in animals includes budding and fragmentation. As a consequence of vegetative reproduction, the microbiome is transferred vertically to offspring. Transmission of microbiota via oocytes and seeds is another example of vertical transmission. Endosymbionts, such as *Buchnera* in aphids [97] and *Wolbachia* in many insects [98], are transferred vertically via oocytes. Vertical transmission in plants has been shown to occur via seeds in many species of herbaceous flowering plants [99], suggesting that this may be a widespread phenomenon.

In vegetarian or omnivore animals, eating mother’s feces (coprophagy) is practiced by many young animals, thereby obtaining the bacteria required to properly digest complex polysaccharides found in their diet. Koalas use a special adaptation of coprophagy [100]. From birth to about 6 months, the joey remains in the pouch, relying only on the mothers’ milk. At the end of this period, the mother produces a liquid form of feces, referred to as pap, which the joey ingests over several days. The pap contains the appropriate gut microbiota for digestion of eucalyptus leaves, enabling eventual weaning from the mother. In the termite hindgut–microbiota symbiosis, feces of adult termites are fed to newly hatched juveniles by workers in the colony [101]. Many insects lay eggs in their feces, which are consumed by larval offspring upon hatching [102]. Depending on the extent that the feces mix with microbes in the environment, transmission by coprophagy can be vertical (e.g., koala) or both vertical and horizontal.

Although it has been reported that the human fetus contains bacteria [103, 104], a recent study using very careful methodologies [105] did not find microbial DNA in the human placenta, suggesting that the human placenta and fetus are sterile. Also, the ability to raise Cesarean-derived germ-free animals in the laboratory argues against a microbiota colonizing the placenta and fetus [106].

Colonization of the newborn human gut occurs initially via inoculation with maternal vaginal and fecal microbes when the baby transits the birth channel (vertical transmission). Some of these pioneers are facultative anaerobic bacteria, such as *Escherichia coli*, which convert the newborn aerobic gut to anaerobiosis, allowing growth of strict anaerobes [107].

Breastfeeding has been shown to provide an additional route of maternal vertical microbial transmission in humans [108–110], nonhuman primates [111], and cows [112]. Human milk contains ca. 10^5^ bacteria per ml, composed of hundreds of species [113]. Analyses of the DNA of several bacterial strains isolated from mothers’ milk demonstrated that they were identical to those found in the offspring [114], providing reinforcement for vertical transmission. Mother’s milk is also a continuous source of modified oligosaccharides that support the growth of the major group of these bacteria, *Bifidobacterium* species, but are not digestible by the infant [115]. The *Bifidobacterium* species contain unique genetic loci responsible for vigorous growth on these oligosaccharides [116]. These findings suggest a remarkable co-evolution between the symbiotic bacteria and their human host enabling gut colonization that benefits both. The *Bifidobacteria* is provided with environmental gains such as food and protection. The infant benefits by being protected against pathogens, by diverse carbohydrate breakdown and cross-feeding activities of the *Bifidobacteria* with other microbes in the gut, thereby enhancing short-chain fatty acid synthesis, and also by specific molecular interactions of the *Bifidobacteria* with infant gut components [117, 118].

Transmission of microbiota from parent to offspring can also occur horizontally, i.e., via the environment. A well-studied example is responsible for maintaining the squid light organ-*Vibrio fischeri* symbiosis [119]. The female host lays clutches of hundreds of fertilized eggs, which hatch almost synchronously at dusk. In parallel, adult squid releases large amounts of *V. fischeri* into the water at dawn every day. The growing embryos develop an immature light organ, which is free of bacteria but has pores leading to separate epithial-lined crypts. These crypts become colonized by the released *V. fischeri*. Furthermore, the developing squid provides a niche in which only *V. fischeri* that emits light is able to maintain a stable association. However, there is no evidence that the *V. fischeri* acquired by the offspring came from one of its parents. It could have come from a different squid in the same environment. Nevertheless, in this horizontal transmission, the holobiont is reconstituted. The reconstruction has to be accurate; otherwise, it does not function [119]. Another example of a reliable horizontal transmission of microbiota is in different kinds of grasses, where the microbes are transferred from one plant to other plants of the same species [120].

As has been discussed above, vertical and horizontal transmissions represent the extreme cases. In most situations, transmission occurs via a mixed mode. Obligatory symbiosis usually relies on vertical transmission while a looser form of symbiosis can be based on a less precise transmission. It is important to note that vertical transmission enables an accurate transfer of the hologenome and ensures continuation of the mutual metabolic activities within the holobiont. Horizontal transfer of microbiota, on the other hand, increases the possibility of the holobiont acquiring novel genetic material. As will be discussed in the next section, acquisition of novel microbes from the environment is one mechanism for gene variation and evolution of holobionts.

Given that several mechanisms exist for the transmission of microbiomes, what is the evidence that microbiomes are actually transferred with fidelity for multiple generations and over evolutionary time scales? One of the first indications that human microbes can be transmitted for many generations came from a detailed analysis of the sequence diversity of DNA isolated from *Helicobacter pylori* present in different geographic human populations [121]. The fact that the distinct sequence remains for centuries in offspring of an individual that has migrated to a different geographical location argues for accurate vertical transmission and has led to the use of *H. pylori* in resolving details of human migration [122]. Another early experiment demonstrating that microbiotas can be maintained for many holobiont generations involved two closely related species of *Hydra* that differed greatly in their bacterial microbiome [123]. Even though these *Hydra* were kept in the same laboratory environment for > 30 years, they maintained their characteristic microbiomes. The authors point out that the microbiotas could have been maintained by vertical transmission or by horizontal selective and differential attachment sites on their membranes.

Long-term transmission of microbiota was studied by comparing the 16S ribosomal gene sequences of bacteria associated with great apes, including humans [124]. The host species phylogenies based on the composition of these microbial communities was completely congruent with the known evolutionary relationships of the hosts. The authors concluded that over evolutionary timescales, the composition of the gut microbiota among great ape species is phylogenetically conserved and has diverged in a manner consistent with vertical inheritance.

However, Moran and Sloan [125] correctly pointed out that vertical transmission of bacterial species, based on 16S rRNA gene sequences, cannot be used to prove co-evolution because it is possible that over evolutionary timescales other strains of the same species (97% identity in 16S rDNA sequence) could be acquired from the environment. To overcome this problem, Sanders et al. [126] developed an elegant analytical tool, beta-diversity clustering, which distinguishes between shared evolutionary history and environmental filtering. The basic idea is that in the case where co-diversification is the primary factor leading to similarity among microbiomes, recent host speciation should be reflected by recent symbiont speciation. By contrast, in the case where host environment selects for different microbes, the most recent common ancestor of a pair of microbes in the two hosts may far pre-date the last common ancestor of the hosts. Using beta-diversity clustering on the previously published great ape data [124] led to the conclusion that apes acquire species-specific microbiota largely horizontally, while retaining a proportion of vertically transmitted microbes over longer timescales. Application of this test to turtle ants (genus *Cephalotes*) indicated a high degree of partner fidelity in the ant microbiota, suggesting that vertical transmission of the entire community could play an important role in the evolution and maintenance of the association [126].

Moeller et al. [127] used rapidly evolving gyrB gene sequences in fecal samples from humans, from wild chimpanzees, and from wild bonobos to profile strain diversity within the gut microbiomes of great apes. Unlike 16S rDNA sequencing, this technology allows inference of the phylogenies of closely related bacterial lineages, thereby enabling tests for co-speciation between gut bacteria and the Hominidae. The analysis revealed that strains of the common gut bacteria, *Bacteroidaceae* and *Bifidobacteriaceae*, have been maintained exclusively within host lineages across hundreds of thousands of host generations. Divergence times of these co-speciating gut bacteria are congruent with those of hominids, indicating that nuclear, mitochondrial, and gut bacterial genomes, i.e., hologenomes, diversified in concert during hominid evolution. Gut bacteria therefore are not simply acquired from the environment, but have co-evolved for millions of years with hominids to participate in their development, especially in shaping their immune systems.

Using the honeybee as a model system, with relatively few microbial species though with similarities to mammalian microbiomes, Kwong and Moran [128] have concluded: “Together, these bacteria form a specialized microbial community that has co-evolved and diversified with its bee hosts over millions of years.” The authors mentioned the importance of sociality to the reliable transmission of microbiota in these species and others.

Phylosymbiosis was proposed to describe the eco-evolutionary pattern, whereby microbiomes parallel the phylogeny of related host species [129]. In support of this hypothesis, it was observed that (i) intraspecific microbiota variation is consistently less than interspecific microbiota variation; (ii) congruence analyses of each group’s complete phylogeny and microbiota dendrogram reveal significant degrees of phylosymbiosis, irrespective of host clade age or taxonomy. This is consistent with selection of host–microbiota interactions driving phylosymbiosis; (iii) there are survival and performance reductions when interspecific microbiota transplants are conducted between closely related and divergent host species pairs.

Co-evolution of animal hosts with their microbiome is made possible by creating a homeostatic relationship between the host and the microbiome. This relationship must be based on prevention of pathological effects of the microbiome in addition to control over the composition of microbial consortia together with immune tolerance towards the microorganisms. It has also to include adaptation of the microorganisms to the specific conditions, in or on the host, and a functional integration of the microorganisms within the holobiont.

It is difficult to imagine such co-adaptation that does not involve a core of microorganisms that fulfills these kinds of homeostatic requirements and that is albeit observed individual species variation (see also discussion above: individual variation: core and conserved function).

## Genetic variation and evolution of holobionts

In our original presentation of the hologenome concept of evolution [2], we suggested that genetic variation and evolution occur not only via changes in host genomes but also via changes in microbiome genomes. In addition to the well-recognized modes of genetic variation in all organisms, mutation, sexual recombination, chromosome rearrangement and epigenetic changes, we considered three underappreciated modes of genetic variation that are characteristic of microbiomes in holobionts: (i) amplification or reduction of the number of a specific microbial group, (ii) acquisition of novel microbes, and (iii) horizontal gene transfer (HGT).

### Amplification or reduction

Amplification/reduction refers to the increase or decrease of one group (e.g., species) of symbionts relative to others, which can occur rapidly when conditions change. The holobiont is a dynamic entity with certain microorganisms multiplying and others decreasing in number as a function of local conditions within the holobiont. An increase in the number of a particular microbe is equivalent to amplification of a whole set of genes. Considering the large amount of genetic information encoded in the diverse microbial population of holobionts, often more than in the host genome [10], microbial amplification/reduction can be a powerful mechanism for contributing to adaptation, development, and evolution of holobionts. Reported examples of environmental factors that lead to changes in symbiont populations and thereby to variations in hologenomes include nutrient availability [130–134], artificial sweeteners [36, 135], food emulsifiers [136], disease states [137–140], pH [141], temperature [142], and of course antibiotics [143–145]. Prebiotics, food ingredients that induce the growth or activity of beneficial microorganisms [146], is an applied example of the amplification principle [147].

Since genetic variation by amplification is driven by the environment, it has a Lamarckian aspect to it, as discussed by us in a previous paper [148]. As will be reviewed in the next section, amplification is a crucial step in genetic variation and evolution by acquisition of novel microbes. For a pioneer microbe to become established in its host it must multiply.

### Acquisition of novel microbes from the environment

Microbes were on this planet for 2.1 billion years before there were any animals or plants. During this time, they evolved enormous biochemical diversity and split into two domains, Bacteria and Archaea. The first eukaryote was probably formed by the acquisition of bacteria to eventually form mitochondria [149] and chloroplasts [150] and possibly by the uptake of an Archaea by Bacteria to form the nucleus [151]. Uptake of microbes into multicellular organisms continued to provide genetic variation for holobionts throughout evolution. Many of the beneficial interactive fitness traits of holobionts discussed above fit into this category.

Animals and plants come into random contact with billions of microorganisms during their lifetime, via air, water and interaction with organic and inorganic surfaces. Occasionally some of these microbes will find a niche and under appropriate conditions amplify in the host and affect the phenotype of the holobiont. Unlike mutation, which causes small changes in existing genomes, acquisition of a microbe introduces hundreds of new genes into the holobiont. Rather than reinvent the wheel, animals and plants can acquire pre-evolved genetic information in the form of microbes. It is likely that after the microbe is acquired, mutations and selection occur in the microbe and host to optimize the interaction.

An example of a major evolutionary event that was driven by the acquisition of bacteria is the ability of many animals to use plant material, in the form of cellulose and other complex polysaccharides, as nutrients. However, animal genomes do not contain the information for synthesizing enzymes for degrading cellulose. Instead, animals such as termites, cockroaches, and ruminants rely on cellulolytic microorganisms that are present in their digestive tract. These microbes anaerobically convert cellulose to fatty acids that are the major source of carbon and energy for their host animal [134]. How did they gain access to these specialized microbes? It is likely that the evolution of termite and cockroach hindgut microbiotas occurred by the gradual process of internalizing from the environment microorganisms that digest plant litter. Instead of plant cellulose being broken down in the soil prior to ingestion, it “rots” in the hindgut after consumption [152]. It has also been suggested that cockroaches acquired cellulolytic microbes by eating the dung of dinosaurs [153], which are known to have been hindgut fermenters [154].

There are many other examples of important evolutionary events that were driven by the acquisition of microbes by animals and plants. Such examples include acquisition of zooxanthellae by corals and other marine invertebrates, which formed photosynthetic animals and led to the construction of coral reefs [155], acquisition of diverse chemosynthetic bacteria by deep sea animals, which allowed for life in the absence of light [156], acquisition of anaerobic bacteria by the gut of ants, which supported herbivory [157], and acquisition of nitrogen-fixing bacteria by legumes, which permitted plant growth under limiting nitrogen conditions [158].

### Horizontal gene transfer

Another important mode of genetic variation in holobionts, referred to as horizontal gene transfer (HGT) or lateral gene transfer, involves the transfer of groups of genes between bacteria of different taxa and from microbiomes to their hosts. HGT is generally associated with gene transfer between different bacteria, but can also take place from microorganisms to animals and plants and the other way around. The intimate contact between microbes and host genomes in holobionts would promote HGTs [159]. On average, bacteria in the human gut contain a minimum of 49 observed horizontally acquired genes [160]. It has been suggested that nutritional adaptation is one of the key selective pressures on the microbiome in the mammalian gut and that HGT processes contribute to that adaptation [161].

An interesting example of evolution of humans by HGT between bacteria is the ability of Japanese to break down agar (an abundant ingredient in their diet) since they have a bacterium in their gut that contains genes that code for the porphyranases that degrade the polysaccharide agarose of agar. Westerners lack this bacterium in their gut and therefore cannot digest agar. The group of genes coding for agarose digestion was driven into a resident gut bacterium by HGT from a marine bacterium that was present on raw seaweed [162]. Although HGT usually occurs between bacteria in the same ecological niche, apparently the marine bacterium was present in the gut long enough to have some of its genes transferred to a resident gut bacterium. Furthermore, the bacteria with the transferred genes spread throughout the Japanese population by vertical and horizontal transmission [163].

Until 2010, only a few examples of HGT from microbes to animals were recognized: *Wolbachia* genes to the chromosomes of their insect hosts [164], bacterial and fungal genes into the telomere region of rotifers [165], fungal genes to aphids [166], and cellulose genes from bacteria to nematodes [167]. However, an examination of the recent availability of a large number of high-quality genomes has led to the conclusion that HGT in animals and plants typically results in tens or even hundreds of active foreign genes [168]. Analysis of the 13.7-megabase genome of the extremophile red alga *Galdieria sulphuraria* revealed that 5% of its protein-coding genes were acquired by HGT from bacteria and archaea [169]. Examination of the genomes of 12 *Drosophila* species showed on average 40 foreign genes that had been horizontally transferred from bacteria and fungi [170]. When the *Drosophila* species were placed on a phylogenetic tree, there was a correspondence between the number of HGT events and the length of each branch, suggesting that HGT has occurred throughout *Drosophila* evolution and is likely to be ongoing. This paper also pointed out that HGT events were more frequent in invertebrates than in vertebrates, an observation that may be explained by the closer proximity between invertebrate endosymbiont (intracellular) and host genetic material.

In humans, 145 genes (not present in other primates) were attributed to HGT [170]. These genes are distributed throughout the genome and play a variety of roles, such as amino-acid metabolism (two genes), macromolecule modifications (15 genes), lipid metabolism (13 genes), antioxidant activities (5 genes), and innate immune response (7 genes). Most of the 145 genes identified in the study came from bacteria, but some originated from viruses and yeasts. Analysis of the moss *Physcomitrella patens* identified 128 genes found in land plants but absent from algae [171]. These genes were acquired by HGT from prokaryotes, fungi or viruses. Many of these genes are involved in some essential or plant-specific activities such as xylem formation, plant defense, nitrogen recycling, and the biosynthesis of starch, polyamines, hormones, and glutathione.

A key event in the evolution of placental mammals, including humans, was the acquisition by HGT, from a retrovirus, of the gene coding for the protein syncytin [172]. Initially, the function of syncytin was to allow retroviruses to fuse host cells so that viruses could move from one cell to another. Now, syncytin is necessary for the development of the placental syncytium, the essential part of the mother-fetus barrier. Knockout of syncytin genes in genetically modified mice provided evidence for their absolute requirement for placenta development and embryo survival [173]. Similarly, retroviral-derived molecules appear to have played a crucial function in the generation of the progesterone-sensitive uterine decidual cell, allowing nutrient provision to the developing embryo [174]. These data indicate that the integration of viral DNA into a host genome played a primary role in a major evolutionary leap by enabling growth and maturation of the fetus in placental mammals. In general, it is clear that introduction of genes by HGT into eukaryote genomes has been a major force propelling genetic variation and evolution.

## Role of microbiomes in speciation

Experiments on speciation in animals provide further support for the hologenome concept of evolution. In 1989, Dodd reported that splitting a homogenous population of *Drosophila* and propagating some of the flies on a molasses medium and the others on a starch medium resulted in mating preference [175]. The “molasses flies” preferred to mate with other molasses flies, and “starch flies” preferred to mate with other starch flies. However, the data could not be explained by existing evolutionary theory because the mating preference was too rapid, especially since there was no selection for mating preference. The experiment was considered important because mating preference is an early event in the emergence of new species [176].

Consideration of the hologenome concept led us to hypothesize that changes in the microbiome were responsible for the diet-induced mating preference. To test this hypothesis, flies were treated with antibiotics. The antibiotics abolished the mating preference, suggesting that fly bacteria were responsible for the phenomenon [177]. This was confirmed when it was shown that infecting antibiotic-treated flies with pure cultures of *Lactobacillus plantarum* isolated from starch flies reestablished mating preference. Furthermore, analytical data showed that *L. plantarum* changed the levels of cuticular hydrocarbon sex pheromones emitted by the flies [178]. In general, volatile metabolites produced by animal microbiomes may play an important role in mate-choice recognition and selection [179].

The microbiome can also play a role in post-zygotic reproductive success. When recently diverged wasp species were artificially cross-bred, the hybrids died during the larval stage. However, if the wasps were treated with antibiotics prior to mating, the hybrids survived [180]. The authors concluded: “In this animal complex, the gut microbiome and host genome represent a co-adapted hologenome that breaks down during hybridization, promoting hybrid lethality and assisting speciation.” Similar results were obtained in two house mice subspecies, suggesting that microbiomes could also contribute to reproductive isolation in vertebrates [181].

## Conclusions

There is now considerable evidence supporting the hypothesis that holobionts with their hologenomes can be considered levels of selection in evolution. The first principle we posited, all animals and plants harbor abundant and diverse microbiota, is now supported by abundant data. The second principle, the holobiont functions as a distinct biological entity, and interactions between microbiomes and their hosts affect the fitness of holobionts, has also been largely substantiated. However, the extent to which the microbiota contributes to holobiont fitness and survival varies enormously. The third principle, where genomes of both hosts and a significant fraction of microbiomes are transferred between generations, is the most contentious. Although there is now evidence that in some animals, microbiota can be maintained by vertical transmission for thousands of generations, it is not possible to generalize on these findings. Finally, regarding the fourth principle, molecular studies have demonstrated that genetic variation and the evolution of holobionts involve acquisition of novel microbes and HGT of microbial genes into host chromosomes.

As the evolutionary biologist Elizabeth Lloyd recently wrote [182], “the holobiont with its hologenome is a level of selection since it is an “interactor”, a “replicator”, a “manifestor of adaptation” and a “beneficiary” of the selection process*.*” Evolution proceeds by both cooperation and competition, working in parallel. An initial mathematical model of holobiont evolution has been reported [182]. Future microbiome research should be expanded to include a larger number of different animal and plant holobiont species and the role of protists and viruses in holobionts.
